# Metabolite differences between glutamate carboxypeptidase II gene knockout mice and their wild-type littermates after traumatic brain injury: a 7-tesla ^1^H-MRS study

**DOI:** 10.1186/s12868-018-0473-5

**Published:** 2018-11-20

**Authors:** Wenbo Wu, Siyi Xu, Jialin Wang, Kuiming Zhang, Mingkun Zhang, Yang Cao, Hongqing Ren, Deyou Zheng, Chunlong Zhong

**Affiliations:** 1 Department of Neurosurgery, Ren Ji Hospital, School of Medicine, Shanghai Jiao Tong University, Shanghai, 200127 China; 2 Department of Neurosurgery, Shanghai East Hospital, Tongji University School of Medicine, 150 Jimo Road, Shanghai, 200120 China; 3 Department of Neurosurgery, Tongzhou District People’s Hospital, Nantong, 226300 Jiangsu China; 4 Department of Neuroscience, Albert Einstein College of Medicine, 1300 Morris, Park Ave., Bronx, NY 10461 USA

**Keywords:** Brain edema, Glutamate, Glutamate carboxypeptidase II, Proton magnetic resonance spectroscopy (^1^H-MRS), Traumatic brain injury

## Abstract

**Background:**

Traumatic brain injury (TBI) is a complex condition and remains a prominent public and medical health issue in individuals of all ages. A rapid increase in extracellular glutamate occurs after TBI, leading to glutamate-induced excitotoxicity, which causes neuronal damage and further functional impairments. Although inhibition of glutamate carboxypeptidase II (GCP II) is considered a potential approach for reducing glutamate-induced excitotoxicity after TBI, further detailed evidence regarding its efficacy is required. Therefore, in this study, we examined the differences in the metabolite status between wild-type (WT) and GCP II gene-knockout (KO) mice after TBI using proton magnetic resonance spectroscopy (1H-MRS) and T2-weighted magnetic resonance (MR) imaging with a 7-tesla imaging system, and brain water-content analysis.

**Results:**

Evaluation of glutamate and *N*-acetylaspartate concentrations revealed a decrease in both levels in the ipsilateral hippocampus at 24 h post-TBI; however, the reduction in glutamate and *N*-acetylaspartate levels was less marked in GCP II-KO mice than in WT mice (p < 0.05). T2 MR data and brain water-content analysis demonstrated that the extent of cortical edema and brain swelling was less in KO than in WT mice after TBI (p < 0.05).

**Conclusion:**

Using two non-invasive methods, 1H-MRS and T2 MR imaging, as well as in vitro brain-water content measurements, we demonstrated that the mechanism underlying the neuroprotective effects of GCP II-KO against brain swelling in TBI involves changes in glutamate and *N*-acetylaspartate levels. This knowledge may contribute towards the development of therapeutic strategies for TBI.

## Background

Traumatic brain injury (TBI) remains a public and medical health concern in individuals of all ages and populations. As it involves the body’s most complex organ, TBI is considered a very complex condition. Treatment results usually vary depending on the severity, pathology, mechanism, and features of the injury [1].

Immediately after TBI, there is a rapid and pathophysiological increase in extracellular glutamate (Glu). This causes neuronal damage and leads to motor and cognitive functional deficits. In the central nervous system (CNS), *N*-acetyl-aspartylglutamate (NAAG), a neuropeptide, is released along with Glu. NAAG binds to the presynaptic type III metabotropic glutamate receptor (mGluR3) and limits the further release of Glu. However, NAAG is hydrolyzed rapidly by the enzyme glutamate carboxypeptidase II (GCP-II), producing Glu and *N*-acetyl-aspartate (NAA). NAAG peptidase inhibitors inhibit the GCP-II enzyme and can reduce the concentration of Glu via two main routes: increasing the duration of NAAG activity at mGluR3, and reducing the degradation of NAAG into NAA and Glu, leading to decreased cell death in models of TBI [2]. It has been reported that GCP-II-knockout (KO) mice develop normally, but demonstrate less astrocyte damage and neurodegeneration after TBI [3]. Together, these findings imply that inhibiting GCP-II is a good approach for reducing the secondary neurodegeneration induced by Glu after TBI, but further experimental evidence is required.

The examination of TBI in animals has become easier due to technological advances in small animal imaging techniques. Cytotoxic and vasogenic edema can be examined by T2-weighted magnetic resonance imaging (MRI) [4], while in vivo brain 1H-MRS can provide information about several compounds that show changes after TBI, such as lactate, creatine (Cr), Glu, and NAA [5–7].

In this study, we explored changes in brain neurotransmitters after TBI by using 1H-MRS, and examined the differences in metabolite status between wild-type (WT) and GCP II gene-knockout (KO) mice after TBI. This non-invasive approach is complementary to our earlier microdialysis and histological studies [3, 8, 9]. The purpose of this study was to obtain further evidence of the usefulness of GCP-II inhibition as a clinical approach to treating TBI.

## Methods

### Animals

Male wild-type (WT) and GCP-II-KO mice, aged 8–12 weeks, weighing 25 ± 3 g, (n = 6 per group) were obtained from the Shanghai Research Centre for Model Organisms (Shanghai, China). Using PCR amplification, we had previously confirmed the genotypes of the KO mice and their WT littermates [3]. Food and water were available ad libitum and mice were housed under a 12-h light/dark cycle at a constant temperature (24 °C) and humidity (50%).

Sodium pentobarbital (65 mg/kg intraperitoneally) was used to euthanize the animals after the experiments.

### Controlled cortical impact TBI

A device for producing controlled cortical impact (CCI) (PinPoint™ PCI3000, Hatteras Instruments, Inc., Cary, NC, USA) was used to induce TBI, as described previously [3]. Mice were anesthetized with sodium pentobarbital, 65 mg/kg intraperitoneally, and fixed in a stereotaxic frame. A heating pad was used to maintain the core body temperature at 37.0 ± 0.3 °C, as monitored by means of a rectal probe. A 5-mm-diameter craniotomy window was made, centred 2.0-mm posterior to the bregma and 2.0-mm lateral to the midline, over the right parietal cortex, and care was taken to avoid damaging the dura. We used a 3.0-mm rounded metal rod, positioned vertically to the brain surface, to induce TBI in WT and GCP II-KO mice. The rod was dropped at a speed of 3.0 m/s to compress the brain to a depth of 1.0 mm for a period of 180 ms. Each mouse in each group underwent surgery with the same precise parameters to mimic moderate TBI in humans. The wound was lightly sutured after injury. Sham CCI WT and GCP II-KO groups underwent identical surgical procedures under anesthesia induced in same manner, but did not experience any CCI injury.

### MRI and ^1^H-MRS acquisition

MRI and MRS experiments were conducted at 24 h after CCI in the test and sham-CCI groups. All MR experiments were performed with a 7-tesla Bruker Biospec animal MRI system equipped with a mouse brain coil (Biospec USR70/20, Bruker, Germany). Briefly, the mouse was initially anesthetized in a chamber with 5% isoflurane in oxygen, and anesthesia was maintained using 1.5–2% isoflurane in oxygen air. The respiration rate was monitored throughout the experiment. Body temperature was kept at 36–37 °C by using a water-heated animal blanket.

A relaxation enhancement sequence (echo time [TE], 33 ms; repetition time [TR], 2.5 s), with a field of view of 1.5 × 1.5 cm and a matrix size of 256 × 256 was used to acquire multislice (0.7-mm slice thickness) T2-weighted images of the mouse brain. In three orthogonal planes, high resolution images were used to locate the position and size of spectroscopic voxels. Variable power radiofrequency pulses with optimized relaxation delay, VAPOR, can suppress water signals. Both an outer volume suppression and point-resolved spectroscopy sequence with a spectral width of 3301.06 Hz, 2048 data points, 256 averages, 2.5 s TR, and 20 ms TE, and an 11-min acquisition time were used to obtain MR spectra. In the hippocampal region, an 8-μL (2 mm × 2 mm × 2 mm) volume of interest was positioned so as to include the CA2 and CA3 regions. The acquired spectra were explored using MestReNova software (Mestrelab Research, Santiago de Compostela, Spain). Each free induction decay was manually phased, and baseline corrected. The NAA peak was referenced to 2.02 ppm [10]. We detected and quantified the NAA peak at 2.02 ppm and 2.50 ppm, the Glu peak at 2.3 ppm, and the creatine peak at 3.03 ppm and 3.93 ppm in mouse brains. Because of its relatively stable concentration in the brain, the creatine (Cr) spectral intensity was used as reference for relative quantitation, to monitor metabolic changes more accurately and to reduce individual variations between mice [11, 12].

### Brain water-content

After euthanizing the mice by sodium pentobarbital, their brains were excised and placed on a cooled brain matrix. Thereafter, the cerebellum and brain stem were discarded. The right and left cerebrum were separated and their wet weights (WWs) were determined. The dry weight (DW) was measured after the hemispheres were dried at 80 °C for 3 days. The water content was then measured as a percentage, using the formula (WW − DW)/WW × 100%.

### Statistical analysis

Data are presented as mean ± SD. SPSS software (version 17; SPSS, Chicago, IL, USA) was used for statistical analyses. Group differences were compared by one-way analysis of variance (ANOVA) and post hoc Bonferroni t-tests. Differences were considered statistically significant if the p-values were less than 0.05.

## Results

### ^1^H-MRS examination

**Fig. 1:**
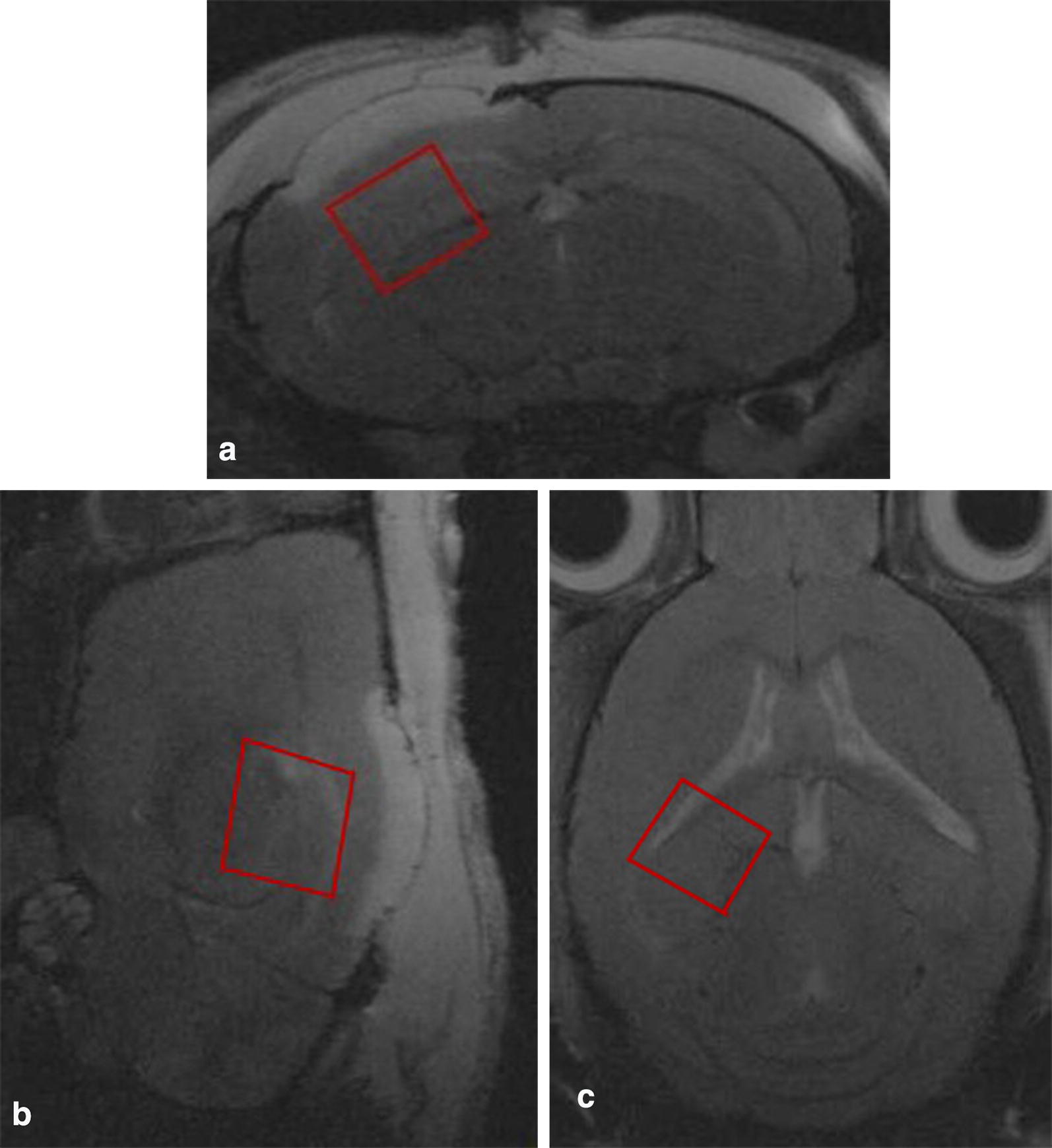
Localized proton MRS of mice brains in vivo (**a** axial, **b** sagittal and **c** horizontal). The ROI was selected in the hippocampus including regions CA2 and CA3 in the right side of the mouse brain, and the ROI sizes were approximately 2.0 mm × 2.0 mm × 2.0 mm

**Fig. 2:**
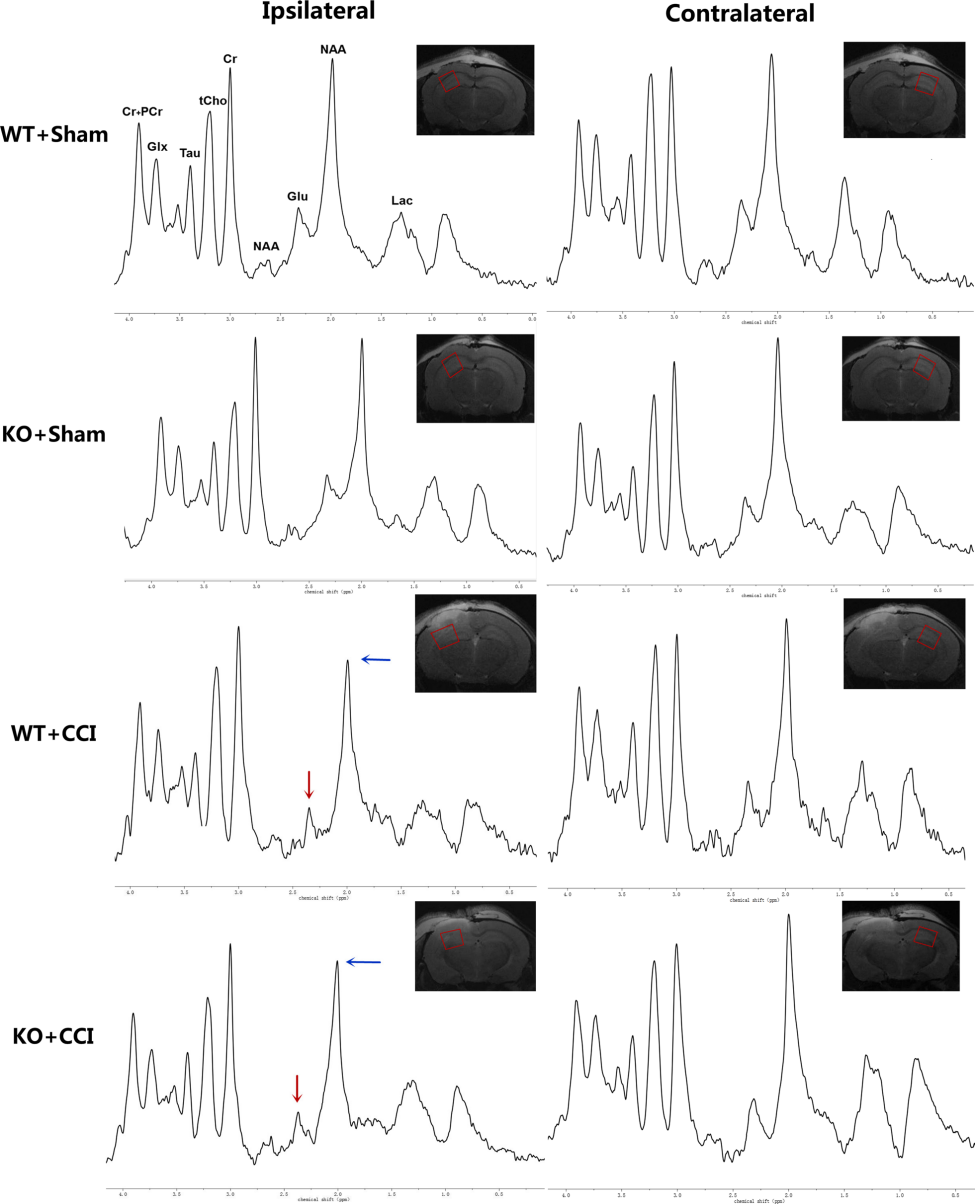
In vivo ^1^H spectra and corresponding voxel location depicted in the anatomic image 24 h post-injury of the GCP II KO mice and there WT littermates. Compared with the bilateral hippocampus of the sham groups and contralateral hippocampus of the CCI groups, the NAA peak (blue arrow) decreased in the ipsilateral hippocampus, as well as the Glu peak (red arrow). And the decrease of NAA peak and Glu peak in KO + CCI group were lower than those in WT + CCI group. *Cr* creatine, *Glu* glutamate, *Glx* glutamine complex, *Lac* lactate, *NAA N*-acetylaspartate, *PCr* phosphocreatine, *Tau* taurine

**Fig. 3:**
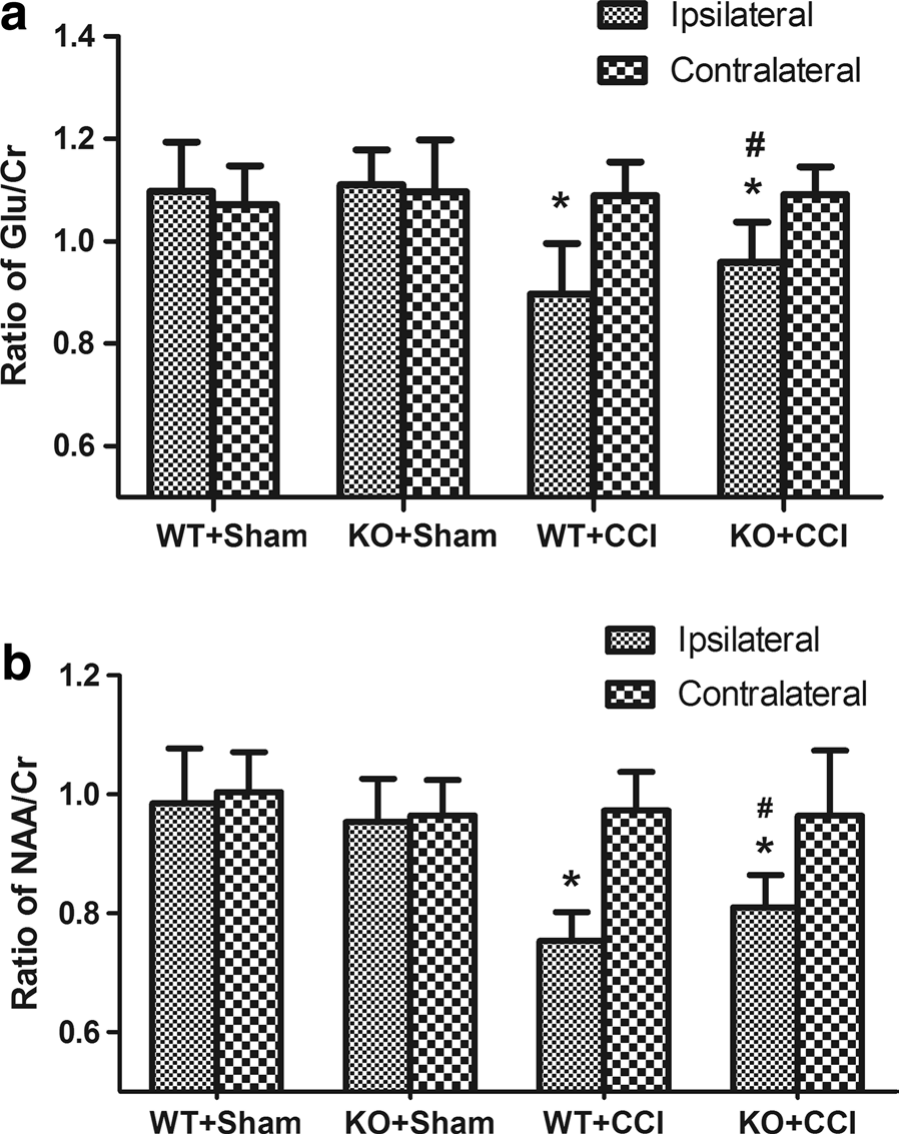
Glu and NAA changes after TBI. **a** The Glu/Cr ratios in the ipsilateral side in both KO + CCI mice and WT + CCI mice were significantly lower than those in the KO + sham and WT + sham mice. This ratio was also significantly lower than that in each contralateral side (*p < 0.05). Moreover, the reduction in Glu/Cr ratio was less marked in GCP II-KO mice than in WT mice (^#^p < 0.05). **b** The NAA/Cr ratio of the ipsilateral hippocampus in GCP II-KO mice was significantly lower than that of the contralateral side and the two sham CCI groups (*p < 0.05). A similar result was found within the ipsilateral hippocampus in WT mice (*p < 0.05), as compared with the contralateral side and the two sham CCI groups. In addition, the reduction in NAA/Cr ratio was less marked in GCP II-KO mice than in WT mice. (^#^p < 0.05)

To measure the alterations in CNS metabolite concentrations after TBI non-invasively, localized 1H-MRS studies were performed on the hippocampal CA2 and CA3 regions. Figure 1 shows the T2-weighted MR image with the MRS voxel position in the hippocampus, while the summed 1H-MRS from the GCP II-KO mice and WT mice are presented in Fig. 2. The concentration of Glu and NAA, referenced to Cr, are shown in Fig. 3. One-way ANOVA showed a significant difference in NAA and Glu levels between groups.

The Glu/Cr ratios of the ipsilateral side in both KO + CCI mice (0.96 ± 0.08) and WT + CCI (0.90 ± 0.10) mice were significantly lower than those in the KO + sham (1.11 ± 0.07) and WT + sham (1.10 ± 0.10) mice. This ratio was also significantly lower than that in each contralateral side (KO + CCI: 1.09 ± 0.05; WT + CCI: 1.09 ± 0.07, respectively) (p < 0.05). Moreover, there was a statistically significant difference in the Glu/Cr ratio of the ipsilateral hippocampus between GCP II-KO mice (0.96 ± 0.08) and WT mice (0.90 ± 0.10) (p < 0.05). The NAA/Cr ratio of the ipsilateral hippocampus (0.81 ± 0.05) in GCP II-KO mice was significantly lower than that of the contralateral side (0.96 ± 0.10) and the two sham CCI groups (0.98 ± 0.09, 0.95 ± 0.07, p < 0.05). A similar result was found within the ipsilateral hippocampus in WT mice (0.75 ± 0.05) (p < 0.05), as compared with the contralateral side and the two sham CCI groups. In addition, the NAA/Cr ratio of the ipsilateral hippocampus in KO + CCI mice was significantly higher than that in WT + CCI mice (p < 0.05).

### T2-weighted magnetic resonance imaging

**Fig. 4:**
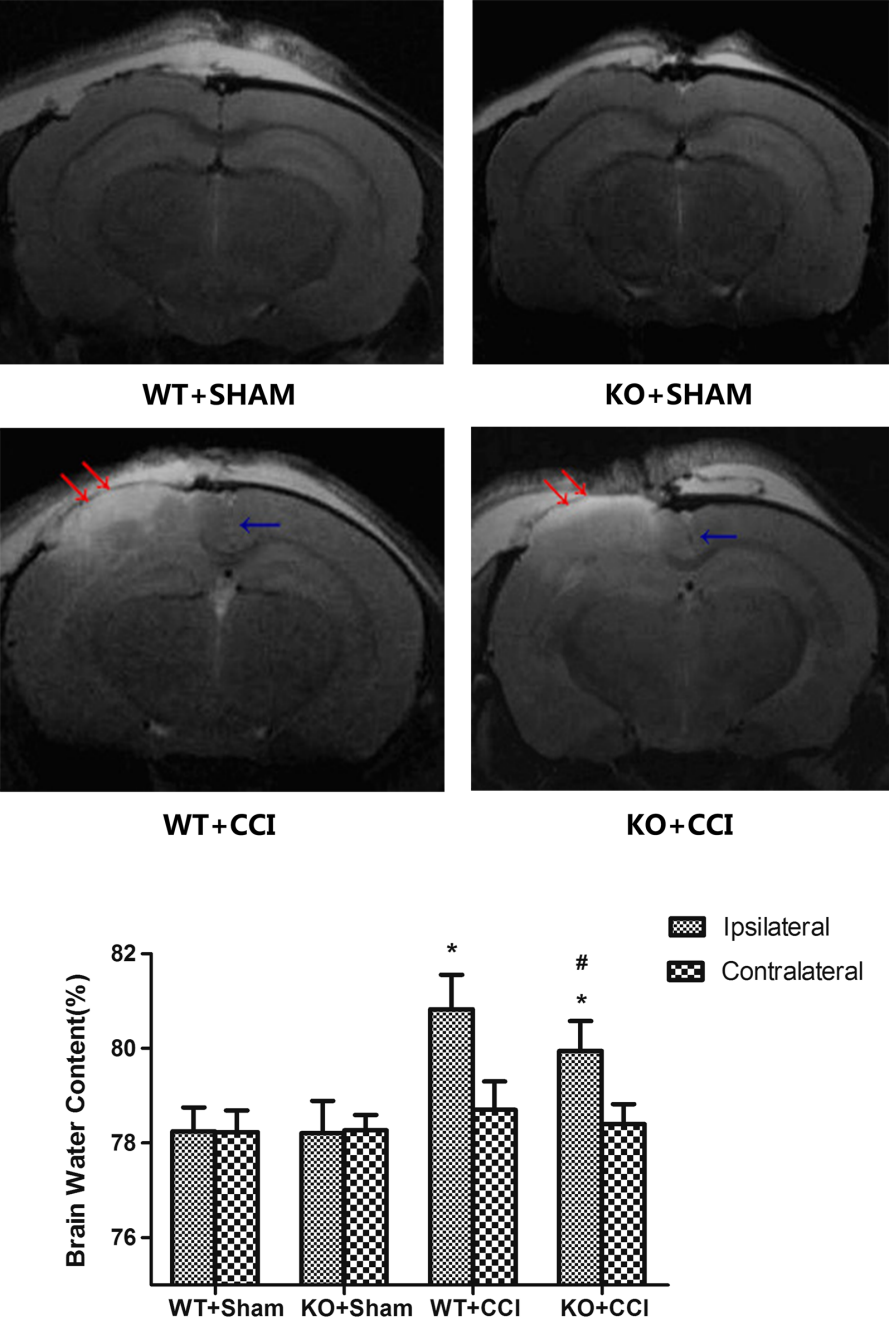
T2-weighted magnetic resonance imaging (MRI) of a mice brain after controlled cortical impact (CCI). Representative coronal images of cortical contusion show that the ipsilateral cortical edema was visible 24 h after injury, as a diffuse tissue hyperintensity (red arrows), and brain swelling was indicated by a midline shift (blue arrow). The extent of both cortical edema and brain swelling in KO + CCI mice was lower than that in WT + CCI mice. The water-content of the contralateral cerebral hemisphere was low and was similar in all groups (p > 0.05). Moreover, the KO + CCI group had a significantly lower brain water-content than did the WT + CCI group (^#^p < 0.05)

The tissue impact of CCI was verified by T2-weighted MRI, which allowed us to observe the brain contusion in vivo (Fig. 4). In the ipsilateral cortex, 24 h after CCI, edema was identified as diffuse hyperintensities. The extent of both cortical edema and brain swelling, indicated by displacement of the midline and the cortical surface towards the contralateral hemisphere, was less in KO + CCI than in WT + CCI mice.

### Brain water-content

To verify the different outcomes of TBI in KO + CCI mice and WT + CCI mice in T2 images, the brain water-content was measured after 1H-MRS; this value was significantly greater in the cerebral hemisphere ipsilateral to the injury in CCI animals than in sham CCI animals (p < 0.05, Fig. 4). The water-content of the contralateral cerebral hemisphere was low and was similar in all groups. Moreover, the KO + CCI group had a significantly lower brain water-content than did the WT + CCI group (p < 0.05, Fig. 4). Therefore, the neuroprotective effect of GCP II-KO against CCI was verified in this study.

## Discussion

After TBI, individual neurochemical changes can be used to elucidate the specific cellular mechanisms involved in TBI. Additionally, MRS can indicate the intracellular regions of interest directly. Thus, this approach can provide information complementary to that obtained by using microdialysis probes, and does so non-invasively. Using 1H-MRS, T2-weighted images and brain water-content analysis in this study, we attempted to determine whether the previously noted protective effect of GCP-II-KO against TBI may be caused by neurometabolic alterations [13]. We therefore investigated Glu and NAA changes in the hippocampus after CCI, using high-field 1H-MRS in vivo. Both Glu and NAA levels in the ipsilateral hippocampus were significantly lower at 24-h post-TBI in GCP II-KO mice than in WT mice (p < 0.05). However, the reduction in Glu and NAA levels was less marked in GCP II-KO mice than in WT mice (p < 0.05). We also showed that the extent of both cortical edema and brain swelling was lower in KO + CCI than in WT + CCI mice, as was the brain water-content.

In the CNS of mammals, Glu is the most abundant excitatory neurotransmitter. Rapid neural depolarizing and release of the excitatory neurotransmitter Glu induced by CCI can lead to overstimulation of Glu receptors, which initiates neuronal damage. Therefore, we considered that 1H-MRS measurements of Glu may provide an in-depth understanding about excitotoxicity after CCI.

At the very early stage of TBI, changes in the concentration of Glu may indicate an imbalance in excitatory and inhibitory activities in the hippocampal region. Our previous studies had shown that the GCP-II inhibitors can, to a certain extent, reduce the release of Glu into the extracellular fluid, with an increase in NAAG levels in animal models that have undergone moderate TBI [8, 9]. Previous 1H-MRS studies have suggested a decrease in the Glu/Cr ratio in the hippocampal region of rats at 2 h and 4 h after CCI, which was deemed to be associated with the glutamate–glutamine cycle and the increased energy requirement of the hippocampal neurons after trauma [14, 15]. We found a decrease in the Glu/Cr ratio at 24 h after CCI in GCP II-KO and WT mice, which indicates that the CCI-induced decrease in Glu levels may contribute to neurological dysfunction in the long run. Concurrently, the less marked reduction in Glu levels in the ipsilateral side of GCP-II-KO CCI mice than in that of WT CCI mice demonstrated that TBI had a less marked effect on mice lacking the GCP-II gene. We hypothesized that NAAG persisted in the synaptic septum after TBI in mice lacking the GCP II gene, inhibiting further release of Glu from the presynaptic membrane, and slowing the deterioration of hippocampal neurons after TBI. Our finding of a decline in Glu levels by 24 h after CCI is consistent with those of a similar study [15].

In the adult brain, NAA is the second-most abundant amino acid. Little is known about the function and metabolism of NAA, even though it is one of the key organic constituents of the brain [16]. Because NAA is found almost exclusively in neurons [17], neuronal injury and death in various diseases and injuries are reflected by the reduction of NAA [18, 19]. The noteworthy decline in the NAA/Cr ratio by 24 h after CCI is in accordance with the findings of other studies that have used similar approaches [15]. In addition, after TBI, the prolonged decline in NAA can lead to diffuse axonal injury or neurodegeneration [20]. We found that the marked decrease in NAA by 24 h after injury in the ipsilateral hippocampus region of KO CCI mice was statistically significantly less than that observed in WT mice. Although the function of NAA is not yet fully understood, it is considered to be a biomarker reflecting neuronal mitochondrial status [20]; consequently, the decrease may be due to impaired NAA synthesis by the mitochondria [7, 15, 21]. Several studies have provided evidence to support the view that a reduction in NAA reflects irreversible neuronal/axonal loss, and is caused by defective NAA synthesis in the mitochondria [22, 23]. Moreover, it has been found that the decrease in NAA in diffuse TBI is partly accompanied by an ATP decline, and recovers only with the restoration of ATP levels [24].

Immediately after CCI, a depolarizing cascade rapidly and simultaneously releases excitatory neurotransmitters, including Glu and NAAG, into the extracellular fluid. At the mGluR3, the escape of abundant excitotoxic Glu is partially inhibited by NAAG, which acts as a potent agonist of the receptor. However, the released NAAG is rapidly inactivated by GCP-II. Previous studies have reported that inhibition of GCP-II plays an endogenous protective role by increasing extracellular levels of NAAG, and that inhibiting GCP-II may be an important approach to increasing extracellular NAAG. Our recent study showed that GCP-II-KO exerts a neuroprotective effect against TBI by preserving mitochondrial integrity in the ipsilateral cortex [25]. It is possible that GCP-II-KO mitigates CCI-induced ipsilateral hippocampal mitochondrial dysfunction, which would exacerbate the consumption of Glu and inhibit the production of NAA due to a lack of ATP.

We found relatively mild brain edema in the ipsilateral hemisphere of GCP-II-KO mice based on T2 images and the measurement of the brain-water content (Fig. 4). After TBI, the disrupted blood–brain barrier allows water to move to the extracellular compartment (vasogenic edema) from the vessels, which is followed by cellular water uptake (cytotoxic edema/cell tumefaction/swelling). Cytotoxic edema particularly affects astrocytes, as these cells are 10 times more numerous than neurons. The mechanisms underlying the tumefaction remain unclear. It has been commonly believed that direct cell damage contributes to the consumption of ATP. To maintain volume homeostasis, brain cells exude organic osmolytes, including Glu [24]. This requires energy consumption and changes in the transmembrane ion gradients, leading to neuronal depolarization, which induces an increased influx of Na^+^ and Ca^2+^ into the cell. The concomitant energy failure injures the Na^+^/K^+^ ATPase pump system, and the Na^+^ overload cannot be compensated. Consequently, water enters into the cell and the cell volume increases [26].

Our research had some limitations. The injured cortical areas were not included in the study because of unstable detection results. The relationship between additional metabolites and TBI remains to be studied in future. Despite this, 1H-MRS is becoming increasingly valuable for examining human survivors of TBI and for improving novel therapies. Nevertheless, further studies are necessary to elucidate how the neurochemical status observed by 1H-MRS relates to the pathological mechanisms associated with TBI.

## Conclusion

In summary, this study provided neurochemical evidence contributing to an understanding of the mechanism underlying the neuroprotective effects of GCP II knockout against TBI. Further studies are needed to explore the potential changes in neuronal cells during the early period (1 h) after TBI. This may help to clarify the details of the underlying neurochemical changes that may have a critical function in the initiation and progression of TBI-induced disorders, and may help in the development of a strategic plan to alleviate the progression of these disorders.

## Abbreviations

1H-MRS: proton magnetic resonance spectroscopy
CCI: controlled cortical impact
CNS: central nervous system
Cr: creatine
DW: dry weight
GCP II: glutamate carboxypeptidase II
Glu: glutamate
KO: knockout
MR: magnetic resonance
MRI: magnetic resonance imaging
NAAG: *N*-acetyl-aspartylglutamate
TBI: traumatic brain injury
WT: wild-type
WW: wet weight
